# Choice of Analgesia in Patients with Critical Skeletal Trauma

**DOI:** 10.7759/cureus.4694

**Published:** 2019-05-17

**Authors:** Abrar H Khosa, Haq Dad Durrani, Wafa Wajid, Maria Khan, Muhammad Irshad Hussain, Imran Haider, Mahrukh Gulnaz, Shahla Butool

**Affiliations:** 1 Critical Care, District Headquarter Teaching Hospital, Dera Ghazi Khan, PAK; 2 Anesthesiology, D.G Khan Medical College, Dera Ghazi Khan, PAK; 3 Internal Medicine - Critical Care, District Headquarter Hospital, Dera Ghazi Khan, PAK; 4 Internal Medicine, Dow University of Health Sciences, Karachi, PAK; 5 General Surgery, Recep Tayyip Erdogon Hospital, Muzaffar Garh, PAK; 6 Orthopedic Surgery, District Headquarter Hospital, Dera Ghazi Khan, PAK; 7 Critical Care, District Headquarter Hospital, Dera Ghazi Khan, PAK; 8 Internal Medicine, District Headquarter Hospital, Dera Ghazi Khan, PAK

**Keywords:** multi-trauma, polytrauma, pain relief, analgesia, epidural analgesia, systemic analgesia, bupivacaine, opioids

## Abstract

Introduction

The adequate management of thoracic trauma requires a systematic approach including pain control, respiratory therapy, and mobility achieved by surgical fixation. Failure to achieve pain control prolongs hospital stay. There are several options for achieving analgesia including epidural catheters, intravenous narcotics, intercostal, paravertebral or interpleural blocks, oral opioids, or simply a combination of the aforementioned interventions. In this study, we aim to compare the efficacy of thoracic epidural analgesia with systemic analgesia in patients with polytrauma.

Methods

This prospective study was conducted in the intensive care unit (ICU) of District Headquarters Hospital in Dera Ghazi Khan, Pakistan. Patients of age ≥18 years with skeletal trauma - rib fractures, limb fractures, and pelvic fractures - were included in the study. Group A patients were given epidural - bupivacaine and tramadol. Group B patients were given systemic analgesia with intravenous opioids. The severity of pain was assessed on the visual analogue scale (VAS) at time 0, 24 hours, and 48 hours. Data was entered and analysis was performed using Statistical Package for Social Sciences version 22.0.

Results

At 24 hours and 48 hours interval, group A showed a lower mean VAS score than group B (*p *= 0.74; *p *= 0.03). Group A required lesser mean doses of additional short-acting analgesics than group B (4.87 ± 1.06 vs. 6.77 ± 1.44; *p *< 0.0001). In Group A, 94% were discharged and the mortality rate was 6%; in group B, 86% were discharged and the mortality rate was 14% (*p *= 0.21).

Conclusion

Epidural analgesia provides better pain relief and requires fewer short-acting supplementing analgesics as compared to systemic analgesia in patients with multi-trauma.

## Introduction

According to the World Health Organization, trauma is the major cause of mortality among men and women of age 15 to 44 years, with road traffic accidents being the leading contributor to 1.2 million deaths every year [1-2]. Unfortunately, among many other east Mediterranean countries, Pakistan was shown to have the highest death rates from such injuries [3]. Patients with polytrauma present with several injuries such as head injuries, rib fracture, pelvic injury, and long bone fracture. However, chest wall injury has been proven to be the most common and fatal among all the other injuries, making it a significant marker of mortality [4]. In a survey conducted in the United States, half a million patients presented to the emergency departments with thoracic injuries [5]. Pain is the chief complaint among trauma patients. The co-existence of comorbidities and anxiety makes the management of pain more challenging [2].

The adequate management of thoracic trauma requires a systematic approach including pain control, respiratory therapy, and mobility achieved by surgical fixation [5-6]. Such a multi-disciplinary approach has proven better clinical outcomes, particularly for patients older than 65 years. Failure to achieve pain control results in an increased risk of pulmonary infection and prolongs hospital stay [7]. Optimal pain control requires administration of age-specific pharmacological agent, identification of appropriate analgesic intervention, awareness of the adverse effects, and periodic assessment of patients and reevaluating their pain management regimen. There are several options for achieving analgesia including epidural catheters, intravenous narcotics, nerve blocks, oral opioids, or simply a combination of the aforementioned interventions [8]. In this study, we aim to compare the efficacy of thoracic epidural analgesia with systemic analgesia in patients with polytrauma.

## Materials and methods

This prospective study was conducted in the intensive care unit (ICU) of the District Headquarters Hospital in Dera Ghazi Khan, Pakistan after the institutional review board approval, from January 2016 till December 2018.

During the study duration, 71 patients, of age ≥18 years, with skeletal trauma - rib fractures, limb fractures, pelvic fractures - with or without associated injuries such as pneumothorax, hemothorax, soft tissue injury were included in the study. Patients were either postoperative or being conservatively managed. Patients who were critical enough to be on ventilatory support and patients with facial/head trauma were excluded. Patients of the Glasgow Coma Scale (GCS) of ≤8 were also not included as they were not oriented/conscious to understand the study protocol and provide consent. Patients were informed about the study aims in detail and consent was taken. Patients, who refused to participate at the start, or at any point of the study, were also excluded.

Patients were randomized to two groups of analgesia: in group A patients, an epidural catheter was passed using the “loss of resistance” technique via the Tuohy needle. The site of epidural injection was localized as per the injury. Injection bupivacaine hydrochloride of 0.5%, in combination with injection tramadol (dilational technique) as per segment analgesia required, was administered via the catheter. For immediate relief, injection xylocaine of 2% and adrenaline were used intermittently. Group B patients were administered systemic analgesia with intravenous opioids. For immediate relief, short-acting non-steroidal anti-inflammatory drugs (NSAIDs) such as diclofenac sodium suppositories were administered as per patient need. The severity of pain was assessed on the visual analogue scale (VAS) at time 0, 24 hours, and 48 hours. The duration of hospital stay and patient outcome were also recorded for all patients.

Data were entered and analysis was performed using Statistical Package for Social Sciences (SPSS) for Windows, version 22.0 (IBM Corp., Armonk, NY, USA). The mean and standard deviation (SD) were calculated for continuous variables and frequencies and percentages were calculated for stratified variables.

## Results

During the study period, 36 patients were randomized to group A and 35 to group B, after completing the inclusion criteria. In group A, there were 22 (61.1%) men and 14 (38.9%) women. Their mean age was 33.58 ± 9.84 years. There were 19 (52.8%) cases of road traffic accidents, seven (19.4%) cases of assaults, nine (25%) cases of fall, and one (2.8%) case of domestic violence. There were 19 (52.8%) patients with multiple ribs with/without limb fractures, and three (8.3%) patients developed pneumothorax. In 11 (30.6%) patients, the pelvis was included, and in six (16.7%) patients, only limbs were involved. In group B, there were 20 (57.2%) men and 15 (42.8%) women with a mean age of 29.44 ± 8.77 years. There were 16 (45.7%) road traffic accident cases, three (8.5%) occupational injuries, 11 (31.4%) assaults, and five (14.3%) cases of fall. There were 25 (71.4%) patients with one or more limb fractures; out of which, 13 (37.1%) had involvement of ribs. Pelvis was involved in 10 (28.5%) patients. Almost all patients in both groups had associated soft tissue injuries and skin wounds and/or lacerations.

Pain severity was assessed in all patients at three time intervals. Patients of group A had a significantly lower mean pain severity at 46-hour interval. Patients of group A also required lesser doses of additional short-acting analgesics as compared to group B patients. Pain severity, need for short-acting analgesics, and patient outcome of both groups are compared in Table 1.

**Table 1:** Comparison of pain severity, need for short acting analgesics and patient outcome of group A and group B SD, standard deviation; VAS, visual analogue scale Group A, epidural analgesia group; Group B: systemic analgesia group

Variables	Group A	Group B	P value
VAS Score (Mean ± SD)			
At time 0	7.68 ± 2.36	7.04 ± 1.89	0.21
At time 24 hour	5.88 ± 2.04	6.04 ± 2.11	0.74
At time 48 hour	3.12 ± 1.87	4.11 ± 2.06	0.03
Additional doses of short-acting analgesics required over 48 hours (Mean ± SD)	4.87 ± 1.06	6.77 ± 1.44	< 0.0001
Patient outcome (%)			
Discharged	34 (94.4%)	30 (85.7%)	0.21
In-hospital mortality	2 (5.6%)	5 (14.2%)	
Deteriorated/collapsed	5 (13.9%)	10 (28.5%)	0.12

## Discussion

Epidural analgesia provided better pain relief and reduced the need for additional short-acting analgesics as compared to systemic analgesia. There was no effect on the choice of analgesia on patient outcome. The availability of an expert technician to insert an epidural catheter and monitor it during the stay is a major hindrance in adopting this technique.

This study has shown some promising results. However, other forms of analgesia cannot be neglected. Multi-center studies with larger sample sizes, including other trauma as well as other forms of analgesia, must be conducted for more robust results.

Analgesia is one of the most important challenges for physicians. Indication, type, choice of drug, and drug dosage are carefully selected to obtain optimal positive outcomes [9]. Multiple rib fractures (MRFs) are associated with intense pain and life-threatening complications such as atelectasis, pneumonia, consolidation, and respiratory failure, which can ultimately lead to death. This calls for potent analgesic and respiratory care interventions, particularly in the elderly in whom comorbidities interfere with the effectiveness of the preventive measures [10]. Several studies have shown a positive outcome of pulmonary function with earlier epidural pain control interventions [11-12]. However, to date, there is no comprehensive literature that provides a conclusive interpretation of the use of various analgesic interventions.

The cornerstone of thoracic trauma is pain management. Amongst various analgesic options, the two most commonly used and yet the most debated are regional analgesia and intravenous (systemic) analgesia. Regional approach has proven to be more efficacious in elderly (>65 years of age), patients with MRFs, and in patients with severe pain or deteriorating pulmonary function [10]. The commonly used regional techniques include epidural analgesia, paravertebral block (PVB), intercostal, and intrapleural block [13]. Systemic analgesics such as nonsteroidal anti-inflammatory drugs (NSAID), codeine, acetaminophen and opioids are considered when only one or two ribs are fractured [10,14]. However, due to the current opioid endemic, physicians and surgeons are often hesitant to prescribe large amounts of opioids for pain control.

Epidural analgesia is the technique of choice in bilateral rib fracture. In a study conducted by Moon et al., there was a significant reduction in pain and hospital stay in patients receiving thoracic epidural catheters in the epidural space between T5 and T7 rendering it superior over parenteral narcotics [15]. Another article published by Wu CL et al. concluded, by assessing the pain scores, that thoracic epidural analgesia with bupivacaine and fentanyl proved to be a better intervention than intravenous patient-controlled analgesia morphine [16]. Bulger et al. conducted a study in which he provided a definitive association between epidural analgesia and reduced risk of nosocomial pneumonia and shorter duration of mechanical ventilation [17]. In a successful retrospective cohort study by Gage et al., the efficacy of epidural analgesia in reducing both short term and long term mortality was further evaluated [18].

Similarly, Yeh at al. defined the characteristics of thoracic injury associated with beneficial effects of epidural analgesia in patients. In this retrospective study, patients presenting with bilateral rib fractures, more total ribs fractured, and having a longer ICU and hospital stay, were more likely to be affected by epidural analgesia, hence predicting patients with severe thoracic injury will benefit more from epidural analgesia as compared to those treated with IV opioids alone [19]. In another retrospective study conducted in Pakistan, records of 218 patients with rib fractures were reviewed, showing a significant reduction in pain in patients who were adequately managed with epidural analgesia than those receiving intravenous opioids. Such patients also had decreased duration of days spent on mechanical ventilation, ICU, and hospital stay [20].

Likewise, a retrospective cohort study involving 277 elderly patients showed patients treated with epidural analgesia, this group also had an increased incidence of pulmonary complications and longer ICU and hospital length of stay [21]. Kieninger et al. also proposed that patients on epidural analgesia, particularly those with less severe injuries, had a longer duration of hospital stay. The likelihood of respiratory complications was also elevated in these patients. Another interesting proposition made by Kieninger concluded that patients with known cardiopulmonary comorbidities had a poor prognosis when managed with epidural analgesia [22].

Contradicting to previous studies showing the advantages of epidural analgesia, a cohort study by McKendy et al. demonstrated higher overall respiratory complications such as deep vein thrombosis and pulmonary embolism in patients receiving epidural analgesia. The length of hospital stay was also higher in these patients [23]. This research was in coherence with systematic review by Carrier et al., which also found no improvement in mortality, length of hospital and ICU stay, or duration of mechanical ventilation, associated with epidural placement [24]. Epidural blockade can aggravate unstable hemodynamics leading to hypotension, which can be fatal. More importantly, epidural analgesia is contraindicated in patients with the preexisting thoracic spine injury. The risk of developing epidural hematoma and the administration of thromboembolic prophylactic agent for polytrauma patients is another major concern for physicians [25].

## Conclusions

The importance of keeping a multi-trauma patient pain-free cannot be neglected. Adequate analgesia is one of the most crucial aspects of medical management of these patients. Achieving the right balance of analgesics which do not keep the patient too drowsy or create dependence, but also provide pain relief is difficult, at time. If the right expertise is available, then epidural analgesia is a more efficacious choice in patients with multi-trauma.
